# Testing Human Skin and Respiratory Sensitizers—What Is Good Enough?

**DOI:** 10.3390/ijms18020241

**Published:** 2017-01-24

**Authors:** Anki Malmborg, Carl A. K. Borrebaeck

**Affiliations:** 1 SenzaGen AB, Medicon Village, S-223 81 Lund, Sweden; amh@senzagen.com; 2 Department of Immunotechnology, Lund University, Medicon Village (bldg 406), S-223 81 Lund, Sweden

**Keywords:** genomics, skin sensitization, adverse outcome pathways, next generation in vitro tests

## Abstract

Alternative methods for accurate in vitro assessment of skin and respiratory sensitizers are urgently needed. Sensitization is a complex biological process that cannot be evaluated accurately using single events or biomarkers, since the information content is too restricted in these measurements. On the contrary, if the tremendous information content harbored in DNA/mRNA could be mined, most complex biological processes could be elucidated. Genomic technologies available today, including transcriptional profiling and next generation sequencing, have the power to decipher sensitization, when used in the right context. Thus, a genomic test platform has been developed, denoted the Genomic Allergen Rapid Detection (GARD) assay. Due to the high informational content of the GARD test, accurate predictions of both the skin and respiratory sensitizing capacity of chemicals, have been demonstrated. Based on a matured dendritic cell line, acting as a human-like reporter system, information about potency has also been acquired. Consequently, multiparametric diagnostic technologies are disruptive test principles that can change the way in which the next generation of alternative methods are designed.

## 1. Introduction

For more than a decade, regulatory organizations have promoted the development of alternative in vitro methods for assessing chemicals and their effect on humans. The need to fulfill these requirements has urged the larger cosmetic and chemical companies to start refining existing conventional test systems, as well as developing additional approaches. The replacement of animal tests has, however, turned out to be a challenge, and despite significant efforts, only a few ECVAM (European Centre for the Validation of Alternative Methods) endorsed in vitro tests exist. Additionally, no stand-alone assay replacing, e.g., the LLNA (Local Lymph Node Assay), has been achieved. In relation to these single mechanistic tests, the OECD (Organisation for Economic Co-operation and Development) has described the skin sensitization as an adverse outcome pathway and the key events that a reliable test must comprise. Consequently, the prediction of skin sensitization must be able to analyze a number of molecular events and there is now a proposal to form an Integrated Test Strategy (ITS) to bring together available tests, in order to cover these molecular events. This proposal is strongly influenced by the availability of test methods, rather than regarding sensitization as a biological event that should be scientifically analyzed in the most predictive manner [1]. 

The resulting question is whether this strategy is good enough for fulfilling the needs of an alternative approach?

## 2. Genomics and Sensitization

The human genome consists of 20,441 coding genes [2] and is the blueprint of our existence. DNA contains information that regulates all cellular events and ultimately determines human phenotypes. Recent technological advancements have allowed us to interpret this massive amount of information harbored in the genome, and has opened up several intriguing applications, such as face recognition and novel vaccine developments [3]. Furthermore, genomics and gene analysis have established themselves as reliable and predictive sources of information, also within polygenic diseases. Inherent risk prediction used to acquire, for example, predictions of cancer and tumor relapse, have all been proven to be possible, due to multiparametric analysis [4,5,6], generating the necessary information which is unavailable from single biomarker analysis. 

This points to the tremendous information content harbored in DNA, which has the potential to be harnessed in tests predicting allergenicity, like skin and respiratory sensitization. If this can be achieved, this information could be sufficient to predict, not only sensitization risks, but also: (i) the strength of a sensitizer, i.e., potency; (ii) pathways involved in sensitization; (iii) to give mechanistic insights. Consequently, genomics delivers information that cannot be achieved when monitoring only one or two markers, such as DC activation markers (CD86, CD54) [7,8], genes engaged in cytoprotective responses to oxidative stress or electrophilic compounds (*Nrf2*—nuclear factor (erythroid derived 2) like 2; *Keap1*—Kelch-like ECH-associated protein 1) [9,10], and proinflammatory cytokines (e.g., interleukin-18 (IL-18)) [11]. Furthermore, quantitative analysis of covalent binding of electrophilic chemicals to nucleophilic centers in skin proteins, has been utilized to investigate potential skin sensitization [12]. However, all of these approaches provide a rather limited amount of relevant mechanistic data, and consequently present limited information for predicting skin sensitization. 

Genomics, on the other hand, takes advantage of the vast amount of information that can be retrieved from analyzing the transcriptome of cells/tissues/organs, that have been challenged by a particular chemical. By analyzing the transcriptome using genome technologies, such as whole genome microarray technology [13,14], or RNA-seq [15], a holistic view of the biological event lying behind complex processes can be observed, rather than a biological snap shot. Decoding genomic information in silico into knowledge by bioinformatics, focusing on yes/no decision values, i.e., hazard determination [13], strength of sensitization, i.e., potency [16], and complex mechanistic analysis, i.e., pathways [17], opens up an entire novel avenue of possibilities to, not only decipher single molecular events, but to deliver information on the underlying events of the proposed adverse outcome pathways. 

## 3. Design of Next Generation Sensitization Tests

Since the testing of ingredients using animals, e.g., the murine local lymph node assay (LLNA), was banned from the cosmetic industry in 2013, alternative in vitro test systems are very high on the wish list. In the development of these new test systems, the ultimate goal is a highly predictive system for mechanism(s) underlying the human biological event one wishes to classify. The only true predictive model organism for humans, is the human being itself. The closest we can get to a human, is a cell system based on human cells, providing relevant mechanistic data by mimicking relevant biological events. The biological event we wish to classify is skin sensitization, which is an immunological reaction. Consequently, a cell system involved in the immune response to foreign matter should be the obvious choice, since this would generate a read-out as close as possible to a human sensitization reaction. A very central cell in the immune system, orchestrating a variety of different immune processes, such as T cell responses, is the dendritic cell (DC). Consequently, a logical choice is to select a dendritic cell line, in particular one that resembles the in vivo counterparts as closely as possible. In a comparative study of myeloid leukemia-derived cell lines, their ability to serve as in vitro models for dendritic cells was investigated [18]. Their capacity to mature into functional dendritic cells, expressing costimulatory molecules, was assessed by functional and transcriptional profiling, and compared with that of monocyte-derived dendritic cells [18,19]. In particular, the original MUTZ-3 cell line displayed the phenotypic and transcriptional profiles of immature dendritic cells, and matured phenotypically, exhibiting a gene induction similar to that of monocyte-derived dendritic cells. The cellular and transcriptional activity of these mature cells, in response to stimuli, demonstrated the value of such a DC-like cell line as a model of the human immune system. Furthermore, the utilization of a dendritic cell line allows the test to be standardized and the quality to be controlled, in a way that can never be achieved when using, e.g., freshly isolated peripheral blood mononuclear cell (PBMC)-derived DCs. 

In addition to DCs, another important cellular component in the sensitization process is the epithelial cells, which also undergo phenotypic alterations upon chemical stimulation, and consequently could also form the basis for a test, particularly in combination with global genomic or proteomic technologies.

In an attempt to materialize the next generation of multi-mechanistic test principles into a predictable method, the Genomic Allergen Rapid Detection (GARD) [13,14] assay was developed and designed, in order to predict the skin sensitizing capacity of chemicals. In short, a matured dendritic cell line is utilized as a human-like reporter cell line [18], and subsequently exposed to test substances. The assay then monitors changes in the expression of around 200 genes. After the analysis of expression levels, compounds can then be predicted as either sensitizers or non-sensitizers, by a Support Vector Machine model. Due to the high information content, the assay delivers important and novel insights into pathway utilization, as well as the chemical potency. Based on blinded test evaluations, GARD has repeatedly been shown to have an accuracy of around 90%, based on over 100 tested chemicals [13,14,18]. 

Of note, when designing multiparametric genomic test systems based on large biomarker signatures, there are inherent risks in data analysis relating to overfitting and model instability, potentially resulting in an overestimation of test accuracy [20]. To mitigate these risks, independent test cohorts must be used for validation, and the prediction model should be based on appropriate multivariate prediction models, such as support vector machine (SVM), Random Forest, or a multivariate regression [13,14]. 

## 4. Genomics and Test Strategies 

Consequently, by using DNA/mRNA for the prediction of human phenotypes, through the application of methods based on genomics, a tool for investigating in vitro skin sensitization can be provided. Technology has advanced and next generation methods have been developed, currently making it possible to achieve comprehensive predictive assessments of sensitization reactions which cover the events we need to understand, rather than combining traditional single mechanistic methods, in so called Integrated Testing Strategies (ITS). Furthermore, ITS suffers from the inherent uncertainty that the different tests might point in different directions, and there is presently no rational way to deal with this conundrum. However, attempts within the regulatory bodies are being made to formulate guidelines for the handling of this, using concepts such as “2 out of 3” [21]. A Bayesian network based on three validated, single biomarker test systems, chemical parameters, and a quantitative structure–activity relationship (QSAR) model, have also been proposed [22]. However, this further complicates the situation. The controversy surrounding this topic is still severe and the situation remains unclear for the end-user. However, through the use of genomics and the new technology surrounding it, we have the possibility to regard the key events in the adverse outcome pathway leading to sensitization. For example, a genomic analysis of a human-like reporter cell, can provide information on OECD key events 2–4 [23], rather than being limited to a single key event based on the cellular origin of the cell line. The reason for this ability is that genes activated in keratinocytes (*Keap1*/*Nrf2*, aldo-ketoreductase family 1, member C2—*AKR1C2*, IL-18), dendritic cells (cluster of differentiation 86—CD86, CD54), CD34^+^ progenitor cells (C-C motif chemokine receptor 2—CCR2, cAMP-responsive element modulator—CREM), and T cells (IL-2, IL-4), can all be surveyed using a human reporter cell and transcriptional analysis. Consequently, the availability of today’s high-throughput testing approaches [24], originally developed by the pharmaceutical industry [6], have the potential to pave the way for the next generation’s stand alone tests for assessing the risk for skin sensitization. A prediction of which allergens will induce respiratory sensitization, remains a significant challenge, because our understanding of the underlying biological properties is incomplete [25,26]. Of note, a genomic biomarker signature for the classification of respiratory sensitizers was, however, recently identified [27]. Furthermore, the multiple mechanistic insights provided by genomics significantly increase the possibility to assess not only the hazard of a chemical, but also its potency, which is the ultimate goal of in vitro testing [16]. 

Consequently, the answer to the question posed in the title is, “test systems based on few biomarkers with no mechanistic insights are not good enough”, since sensitization should be studied and analyzed as a complex biological event, utilizing test system designs based on such information. 

## 5. Conclusions

In summary, today´s single mechanistic assays have not been proven sufficient as stand-alone sensitization tests, in order to replace those which previously used animals. Consequently, it is imperative that regulatory authorities evaluate and utilise the experience gained during more recent progress in other areas of complex diseases [6], regarding the inadequate value of single mechanistic tests. There is an unprecedented and proven opportunity in multi-mechanistic test strategies, where one genomic tests system has been described above, as an example. This approach represents a shift in the paradigm and, in the near future, will also allow us to predict the potency of both skin and respiratory chemicals in vitro, with higher accuracy.

## Abbreviations

AOP	Adverse Outcome Pathways
DC	Dendritic Cell
PBMC	Peripheral Blood Mononuclear Cells
GARD	Genomic Allergen Rapid Detection
ITS	Integrated Testing Strategy
